# Expression of osteopontin, matrix metalloproteinase-2 and -9 proteins in vascular instability in brain arteriovenous malformation

**DOI:** 10.7717/peerj.7058

**Published:** 2019-06-24

**Authors:** Lalita Anbarasen, Jasmine Lim, Retnagowri Rajandram, Kein Seong Mun, Sheau Fung Sia

**Affiliations:** 1 Department of Surgery, Faculty of Medicine, University of Malaya, Kuala Lumpur, Wilayah Persekutuan Kuala Lumpur, Malaysia; 2 Department of Pathology, Faculty of Medicine, University of Malaya, Kuala Lumpur, Wilayah Persekutuan Kuala Lumpur, Malaysia

**Keywords:** Inflammatory markers, Immunohistochemistry, Intracranial hemorrhage, Arteriovenous malformation, ELISA assay

## Abstract

**Background:**

Matrix metalloproteinase (MMP)-2 and -9 are Osteopontin (OPN) dependent molecules implicated in the destabilization of blood vessels. OPN and MMPs have been studied in brain arteriovenous malformation (BAVM) patients’ tissues and blood samples before intervention. In this study, we compared the serum level of these markers before and after treatment, as well as assessed their protein expressions in BAVM tissues to evaluate their roles in this disease.

**Methodology:**

Serum samples from six BAVM patients and three control subjects were analyzed using enzyme-linked immunoabsorbent assay (ELISA) for OPN. A total of 10 BAVM patients and five control subjects were analyzed using Multiplex ELISA for MMPs. A total of 16 BAVM tissue samples and two normal brain tissue samples were analyzed using immunohistochemistry.

**Result:**

MMP-2 and -9 were significantly higher in the serum of BAVM patients before and after treatment than in control patients. There were no significant differences of OPN and MMP-9 serum level in BAVM patients before and after treatment. MMP-2 showed a significant elevation after the treatment. Expression of OPN, MMP-2 and -9 proteins were seen in endothelial cells, perivascular cells and brain parenchyma of BAVM tissues.

**Conclusion:**

Findings revealed that the level of MMP-2 and -9 in the serum correlated well with the expression in BAVM tissues in several cases. Knockdown studies will be required to determine the relationships and mechanisms of action of these markers in the near future. In addition, studies will be required to investigate the expression of these markers’ potential applications as primary medical therapy targets for BAVM patients.

## Introduction

The prevalence of brain arteriovenous malformation (BAVM) in the general population is approximately 0.001–0.52% (Solomon & Connolly, 2007). According to Kim et al. (2011) BAVM has a slightly male preponderance where the sample population in the particular study was 65.5% males and 34.5% females. Some believe that BAVM are congenital lesions, and therefore the risk of hemorrhage is high and life-long (Komiyama, 2016). Retrospective and prospective observational studies have shown that the estimated risk of spontaneous intracranial hemorrhage (ICH) in patients with BAVM ranged from 2% to 4% per year (Kim et al., 2011).

Brain arteriovenous malformation is one of the major causes of ICH. High blood pressure shunting occurs in the tangle of abnormal vasculature causing weakening of vessel walls which in turn increases the risk of ICH. ICH is an important cause of morbidity and mortality in BAVM (Komiyama, 2016). Pathogenesis of BAVM is still not well-defined. However, some studies revealed that angiogenic factors and inflammatory cytokines contribute towards its initiation and progression (Chen et al., 2008; Kim et al., 2008). The treatments for BAVM are mainly given to reduce the risk of the rupture which could be fatal for patients. The risk of the BAVM to rupture depends on vascular instability. Osteopontin (OPN), matrix metalloproteinase (MMP)-2 and -9 are some of the inflammatory markers involved in pathophysiology of vascular instability. Hence in this study we preliminarily analyzed the expression of these proteins in BAVM patients’ serum samples and compared with controls. We also studied the expression of these markers in tissue samples to analyze the localization of the proteins. This might help in predicting the risk of rupture.

Osteopontin is an acidic glycoprotein which is a member of the small integrin-binding N-linked glycoprotein family of proteins. OPN is also known as secreted phosphoprotein 1, early T-lymphocyte activation 1 protein and bone sialoprotein (Pagel et al., 2014). During inflammation, OPN triggers various leukocytes and evoke functional response and induce secretion of cytokinesis (Castello et al., 2017). A previous study showed that the expression of OPN mRNA and OPN proteins were significantly higher in arteriovenous malformation (AVM) tissues compared to control brain specimens. The study also suggested that OPN might involve vascular instability in BAVM (Xu et al., 2012).

Matrix metalloproteinase is a family of membrane bound and extracellular zinc-dependent endopeptidases involved in matrix degradation and tissue remodeling, around normally and abnormally developing vasculature (Wei et al., 2016). Leukocyte transmigration to the cerebral microvasculature and their accumulation in inflammatory sites in response to cytokines, chemokines, and products of tissue breakdown take place during inflammatory process. In this inflammatory process, leukocytes secrete myeloperoxidase MMPs enzymes and release cytokines including interleukin (IL-6), contributing to brain injury. MMPs are pro-inflammatory cytokines capable of weakening or destroying the vascular wall leading to vascular rupture and hemorrhage (Chen et al., 2008).

Matrix metalloproteinase-2 and -9 are the two main proteases commonly involved and have been studied extensively in tumor metastasis, inflammation, as well as vascular diseases including aneurysms and AVMs (Aoki et al., 2007; Starke et al., 2010). Starke et al. found that MMP-9 is increased in BAVM patients when compared to controls. Immediately after the treatments, embolization or surgery, expression of MMP-9 increased significantly and decreased to the pre-treatment level after 4 weeks (Starke et al., 2010). Activated MMP-9 which stored in tertiary granules of neutrophils, causes hemorrhage in BAVM and acts as an effector as well as regulator of leukocyte function, including maturation of neutrophils (Chen et al., 2008).

It has been shown that OPN, MMP-2 and -9 are implicated in the weakening and destabilization of vessel walls (Jabbour et al., 2009; Solomon & Connolly, 2007). MMP-2 and -9 are OPN dependent molecules.

There is emerging evidence showing the correlation between OPN and MMP-2 and -9. For instance, the down-regulation of siRNA mediated OPN inhibited the expression of VEGF, MMP-2 and -9 and urokinase plasminogen activator (uPA) in Lovo cells (Wu et al., 2014). It has also been shown that increased OPN expression up-regulated the uPA, MMP-2 and -9 via nuclear factor-kB (Mi et al., 2011).

Another study also revealed that cells overexpressing OPN splice variant, OPNa (the full-length variant) secretes extracellular conditioned medium that induces MMP-2 and -9 activities in thyroid cancer (Ferreira et al., 2016).

Currently, there was no documented evidence in PUBMED showing the expression of OPN, MMP-2 and -9 proteins at serum level, before and after treatment. Only few studies demonstrated an increase of MMP-9 protein expression in the endothelial/peri-endothelial cells and infiltrating neutrophils using immunohistochemistry (IHC; Chen et al., 2006; Hashimoto et al., 2003).

## Materials and Methods

### Ethics approval

Ethics approval was obtained from the Medical Ethics Committee of University Malaya Medical Centre (UMMC) for this study (MECID.NO: 201412-888). All samples and data were collected after obtaining written consent from patients and/or their next-of-kin.

### Sample acquisition

#### Tissue

Formalin fixed paraffin embedded (FFPE) samples of radiologically diagnosed BAVM were collected retrospectively (2001–2013) and prospectively (2014–2016) from the archives of Department of Pathology and by UMMC BIOBANK UNIT Faculty of Medicine, University of Malaya. Control samples were brain cortex tissues taken from the uninjured part of the brain from deceased patient with intraparenchymal bleed following skull fracture from motor vehicle accident, and from patient who has died of sepsis from liver abscesses. These control samples were normal brain tissue macroscopically and microscopically within normal limits. Both BAVM and control tissues were fixed in 10% buffered formaldehyde overnight and followed by sampling, processing and embedding into paraffin wax.

#### Blood

For BAVM subjects, blood collection was performed prospectively 24 h before and 2-week after surgery or/and embolization treatment between August 2014 and June 2016. Serum controls were collected from age-matched healthy individuals without radiologically diagnosed BAVM. All the blood samples were centrifuged to separate the serum. Serum was then aliquoted into cryovials and stored −80 °C prior to analysis.

### Immunohistochemistry

#### Antibodies

The antibodies used were mouse monoclonal anti-OPN (clone OP3N, NOVOCASTRA; Leica Biosystem, Newcastle Upon Tyne, UK) and mouse monoclonal anti-MMP-9 (clone 15W2, NOVOCASTRA; Leica Biosystem, Newcastle Upon Tyne, UK), and mouse monoclonal anti-MMP-2 (clone6E3F8, ABCAM; Biotech, Life Sciences, Cambridge, MA, USA). All antibodies were optimized and validated for IHC in previous studies (Li et al., 2017; Perrotta et al., 2011; Sulik & Chyczewski, 2008).

#### Single immunostaining procedure

A total of 16 BAVM and three control FFPE tissue were sectioned into four μm, followed by deparaffinization and rehydration. Incubations were performed at room temperature unless stated otherwise. For antigen retrieval, 15 min of microwave heat treatment was conducted in EDTA buffer (one mM, pH 9) for anti-OPN and citrate buffer (10 mM, pH 6) for anti-MMP-9 as well as anti-MMP-2. Samples were allowed to cool to room temperature before incubation with Novocastra™ Peroxidase Block for 5 min to neutralize endogenous peroxidase activity and Novocastra™ Protein Block to reduce non-specific binding of primary and polymer. Subsequently, sections were incubated overnight at 4 °C with primary antibodies including anti-OPN (1:300), anti-MMP-2 (1:500) and anti-MMP-9 (1:40). For negative controls, primary antibodies were replaced with Tris-buffered saline. Positive tissue controls for anti-OPN, anti-MMP-2 and anti-MMP-9 were colon, neurofibroma and liver, respectively. Primary antibodies were detected using Novolink™ Polymer Detection System (RE 7150-K; Leica Biosystems, Wetzlar, Germany) according to the manufacturer’s recommendations. Staining was completed by a 10 min incubation with 3,3′-diaminobenzidine (DAB+) substrate–chromogen buffer before counterstaining with hematoxylin. The staining was assessed independently by two investigators (LA and MSK) using a systemic semi-quantitative scoring system based on the approximate proportion of cells stained: +++ (nearly all cells stained >80%), ++ (approximately half of the cells stained =50%), + (a low percentage of cells stained <10%), negative (no positive cells detected).

#### Serum analysis by ELISA

A total of nine serum samples (six BAVM patients and three healthy controls) were analyzed. OPN enzyme-linked immunoabsorbent assay (ELISA) kit (Cusabio Biotech Co., Ltd, Wuhan, China) was used according to the manufacturer’s guidelines. Briefly, 100 μL each of standard reagent and serum samples were added together prior to 2 h incubation at 37 °C in microtiter plates with wells coated with OPN antibody. These plates were washed to remove unbound OPN protein before the detection antibody (Biotin antibody) and substrate solution, horseradish peroxidase (HRP-avidin) were added. The color development was allowed for approximately 15 min with 3,3′,5,5′-tetramethylbenzidine substrate. The color development stopped using STOP solution (0.16M sulfuric acid) and the colorimetric density of each well was measured at 450 nm using a microplate reader with standard dilutions set as 50, 25, 12.5, 6.25, 3.12, 1.56, 0.78 and 0 ng/ml. The standard curve was prepared and the values of the samples were calculated. Quantitative comparisons were then made from the duplicated analysis of each patient or control.

#### Serum analysis by Multiplex ELISA

A total of 15 serum samples (10 BAVM patients and five healthy controls) were analyzed. MMP-2 and -9 Multiplex kits were used (EMD Millipore Corporation, Billerica, MA, USA) according to the manufacturer’s guidelines. A total of 25 μL of standards, controls and samples together with 25 μL beads containing MMP-2 and -9 were added and incubated for 2 h at room temperature while agitated on a plate shaker. Then the plate was washed in a magnetic plate washer before incubating with 25 μL detection antibodies and incubated for 1 h while agitated on a plate shaker. Following this, 25 μL Streptavidin–Phycoerythrin were added and incubated for 30 min with agitation on a plate shaker. The plate was again washed on magnetic plate washer before adding 100 μL of sheath fluid which serves as a delivery medium to transport the sample to the optics component of the Luminex xMAP technology-based instrument and resuspended the plates on plate shaker for 5 min. Then, the plate was put through the Luminex 200TM. The median fluorescent intensity data was analyzed using spline curve–fitting method for calculating analytes concentrations in samples.

#### Statistical analysis

The expression levels of OPN, MMP-2 and -9 were presented as the median ± range deviation (SD). The significance differences among the groups were calculated using Wilcoxon signed rank test and Mann–Whitney *U*-test. All statistical analysis was performed using SPSS for Window version 21.0 (SPSS Inc., Chicago, IL, USA). Two-tailed *p* < 0.05 was considered as statistically significant.

## Results

### Expression of OPN in serum of BAVM patients

The blood samples were collected from six BAVM patients 24 h before surgery and 2 weeks after surgery. The mean age for BAVM patients and three controls were 28.0 ± 9.5 and 34.3 ± 8.1 years, respectively (*p* = 0.357; *t*-test). There was no significant difference of the average ages between BAVM patients and controls.

**Figure 1:**
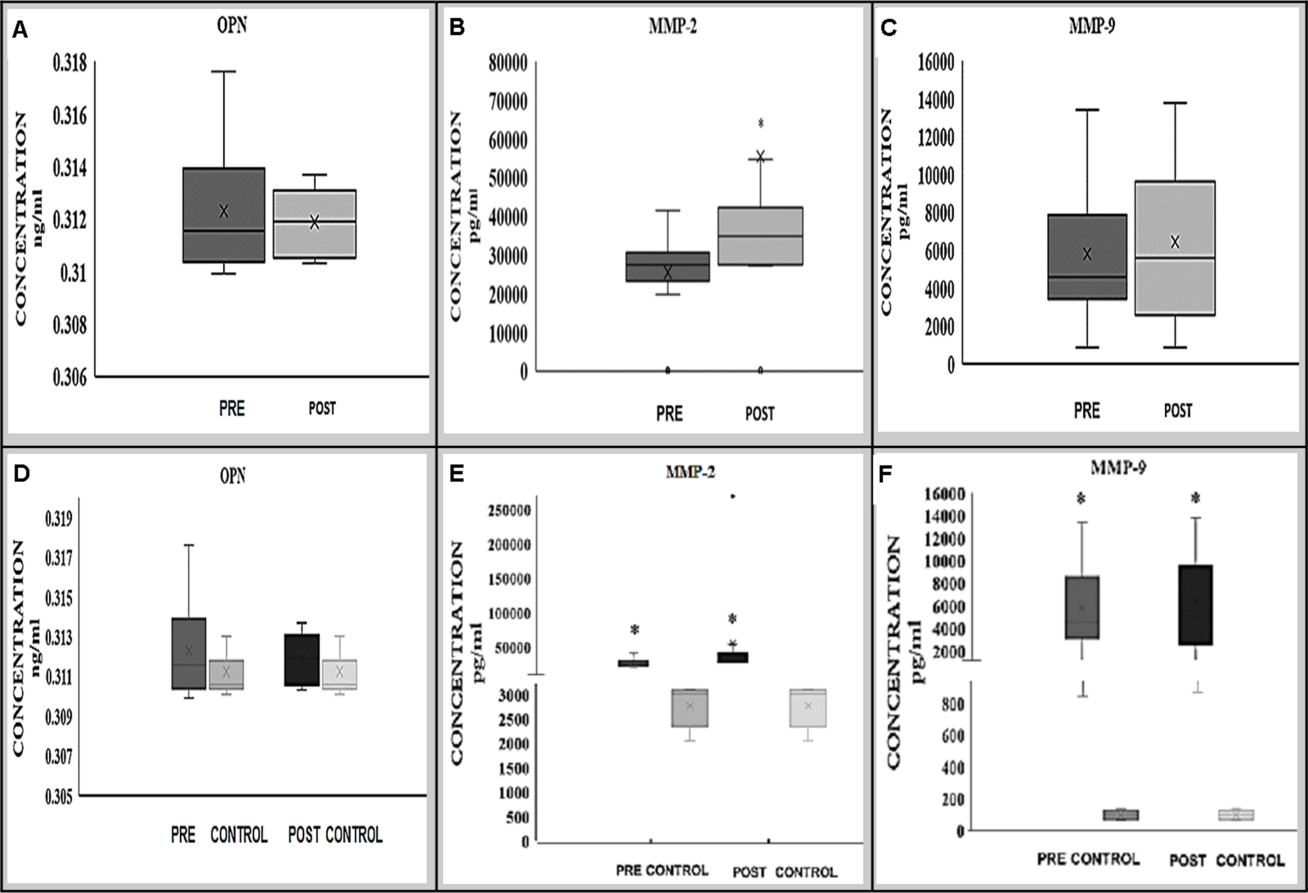
Expression of OPN (A, D), MMP-2 (B, E) and MMP-9 (C, F) proteins in BAVM patients (before and after treatment) alone and in comparison with controls. MMP-2 (B) protein was significantly increased after treatment whilst there was no significant changes of OPN (A) and MMP-9 (C) protein expression before and after treatment. Comparing to the controls, MMP-2 and -9 (E, F) protein expression were significantly higher in BAVM patients preoperatively and postoperatively; however, no significant difference was observed in OPN (D) protein expression.

Preliminary analysis of OPN in BAVM patients showed that no significant differences in the serum OPN level either before (median; range, 0.312; 0.008 ng/ml) or after (median; range, 0.312; 0.003 ng/ml) treatment in BAVM patients (*p* = 0.917; Wilcoxon signed rank test) (Fig. 1A).

There were also no significant differences in the expression of OPN in the serum of BAVM patients from the controls (median; range, 0.311; 0.003) either before (median; range, 0.312; 0.008 ng/ml) or after (median; range, 0.312; 0.003 ng/ml) treatment, with *p*-values of 0.281 and 0.951, respectively (Fig. 1D).

**Figure 2:**
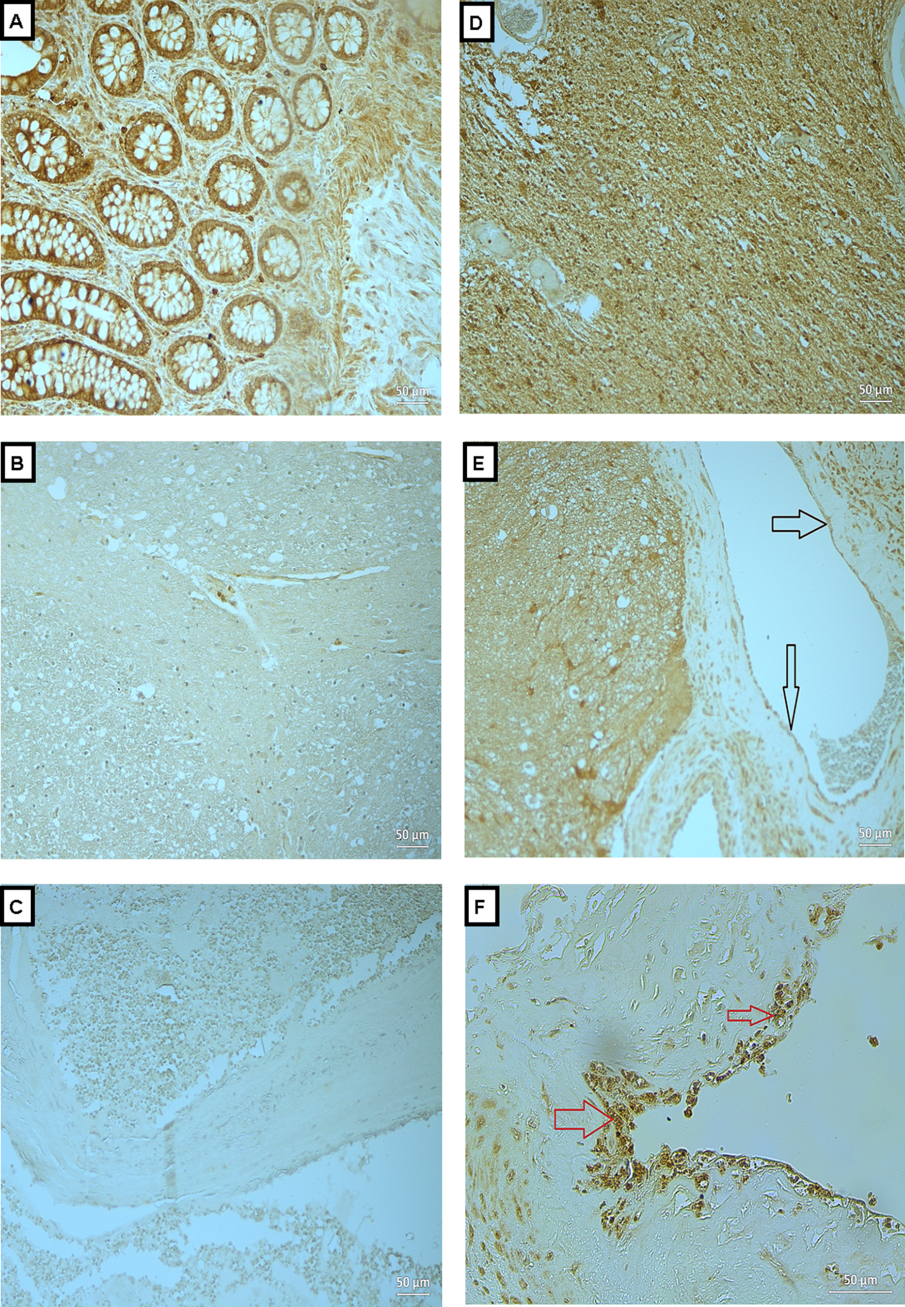
Expression of OPN in BAVM tissues. (A) is a colonic tissue (positive control) showed positive staining in the intestinal glands. (B and C) are normal brain tissue with antibody and BAVM tissue without antibody, respectively, as negative controls, both showed negative staining for OPN. (D) is BAVM tissue stained with OPN, showed positive staining the of the brain parenchyma. (E and F) are also BAVM tissues, showing positive staining of OPN in the endothelial cells (black arrow) and perivascular cells (red arrow), respectively. A total of 16 BAVM tissues were stained with anti-OPN. Original magnification ×200 (A–E) and ×400 (F).

### Expression of MMP-2 and -9 in serum of BAVM patients

Expression of MMP-2 showed a significant difference in the serum sample of BAVM patients 24 h before (median; range, 27,395; 41,350 pg/ml) and after treatment (median; range, 34,950; 269,710 pg/ml), with *p*-value of 0.011* (Wilcoxon signed rank test) (Fig. 1B).

Comparing with control samples (median; range, 3,009; 1,052 pg/ml), MMP-2 expression was significantly higher in BAVM patients preoperatively (median; range, 27,395; 41,350 pg/ml) (*p* = 0.014; Mann–Whitney *U*-test) and postoperatively (median; range, 34,950; 269,710 pg/ml) (*p* = 0.014; Mann–Whitney *U*-test) (Fig. 1E).

Expression of MMP-9 in pre-treatment (median; range, 4,551.0; 12,585.7 pg/ml) serum sample of BAVM patients showed no significant difference when compared with post-treatment (median; range, 5,589.5; 12,939.7 pg/ml) serum samples with *p*-value of 0.575 (Wilcoxon signed rank test) (Fig. 1C). However, when compared to control samples (median; range, 97.3; 65.4 pg/ml), MMP-9 expression was significantly higher in BAVM patients preoperatively (median; range, 4,551.0; 12,585.7 pg/ml) (*p* = 0.002; Mann–Whitney *U*-test) and postoperatively (median; range, 5,589.5; 12,939.7 pg/ml) (*p* = 0.002; Mann–Whitney *U*-test) (Fig. 1F).

### Expressions of OPN, MMP-2 and -9 in BAVM tissues

**Figure 3:**
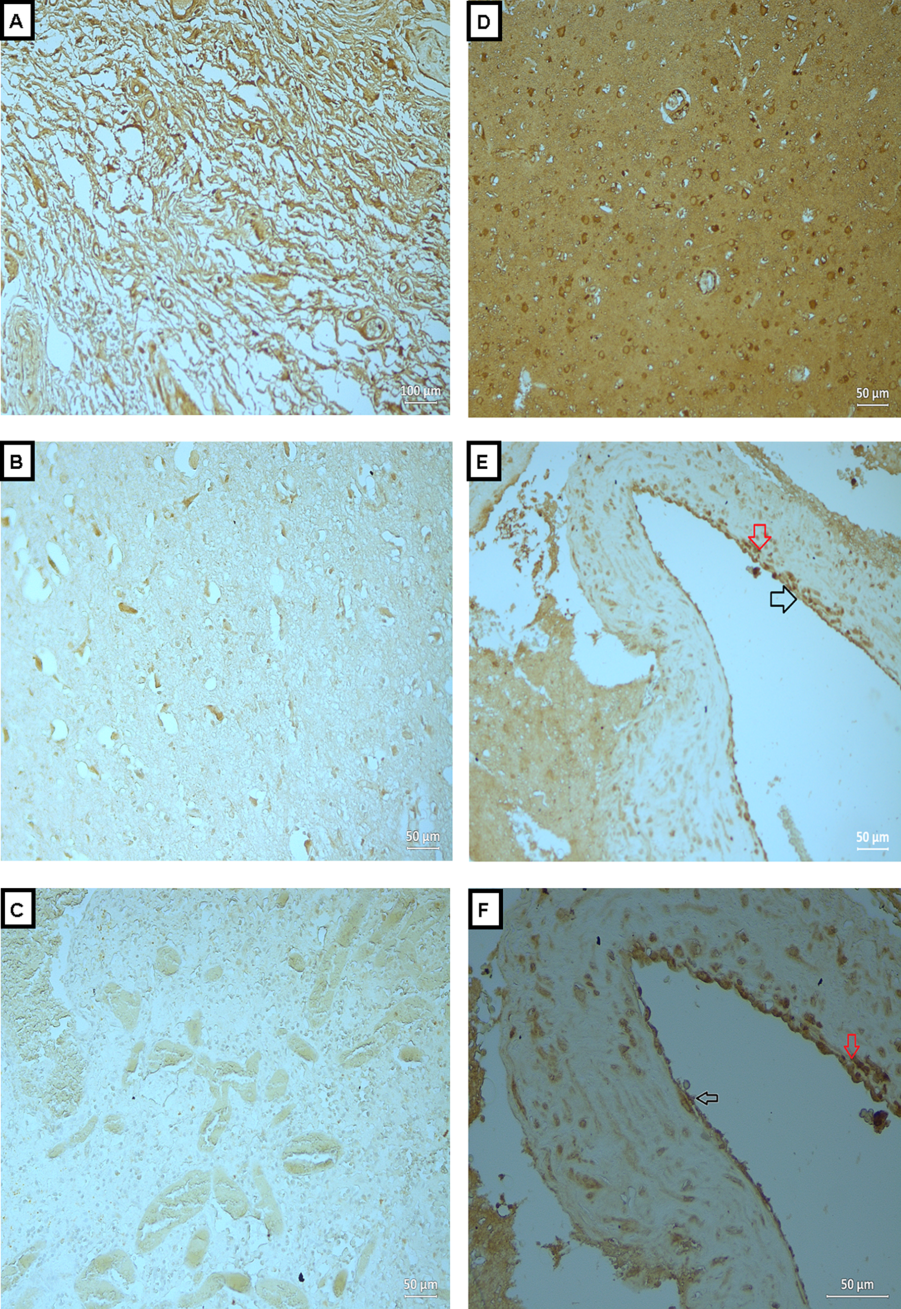
Expression of MMP-2 in BAVM tissues. (A) is section of neurofibroma (as positive control), showed positive staining of MMP-2 in the neural elements. (B and C) are normal brain tissue with antibody and BAVM tissue without antibody, respectively, as negative controls, both showed negative staining. (D) is BAVM tissue, showed positive staining in the brain parenchyma. (E and F) are also BAVM tissues, both of which showed positive staining of MMP-2 in endothelial cells (black arrow) and perivascular cells (red arrow). A total of 16 BAVM tissues were stained with anti-MMP-2. Original magnification ×100 (A), ×200 (B–E) and ×400 (F).

**Figure 4:**
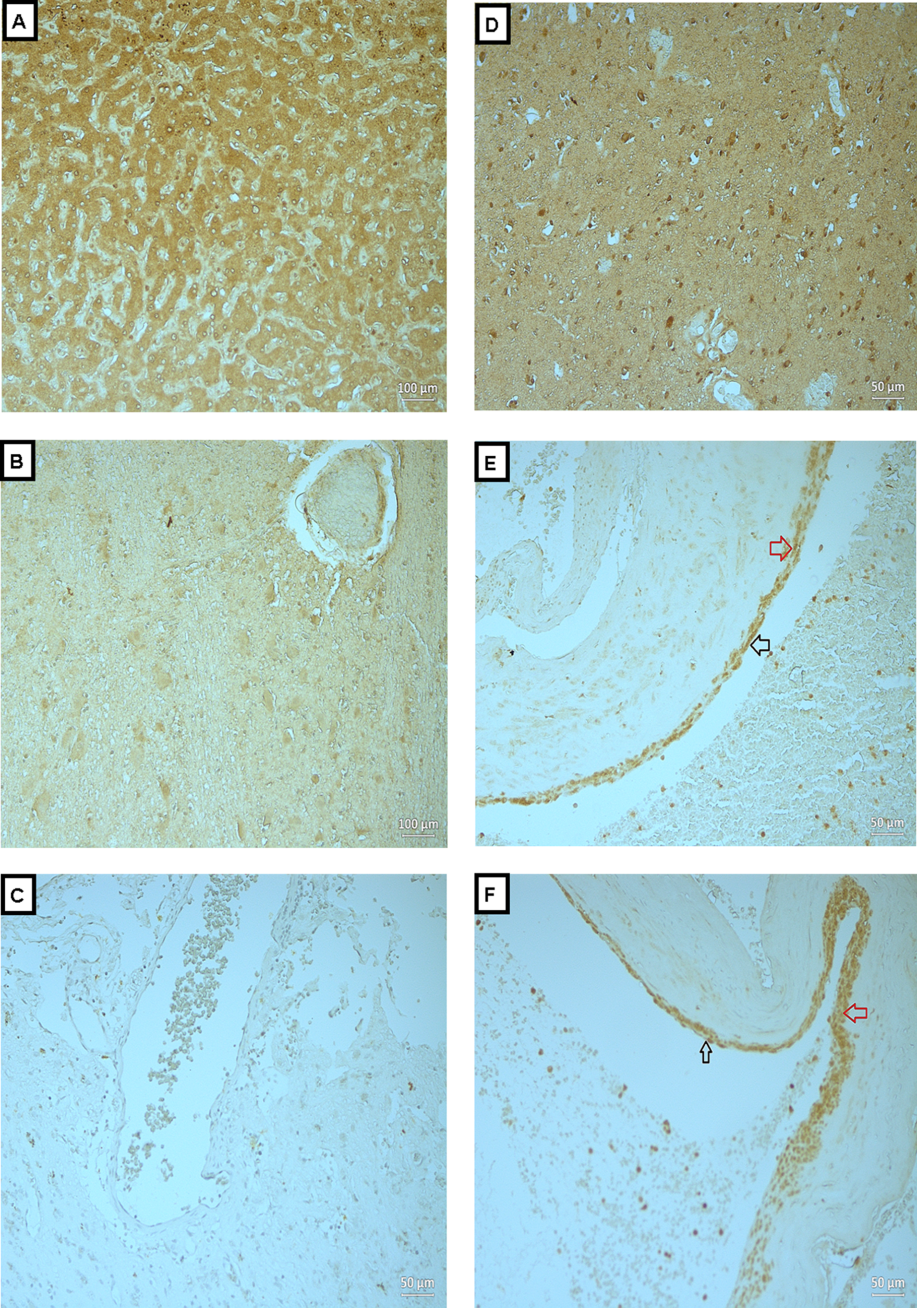
Expression of MMP-9 in BAVM tissues. (A) is liver tissue (as positive control), showed positive staining of MMP-9 in the hepatocytes. (B and C) are normal brain tissue with antibody and BAVM tissue without antibody, respectively, as negative controls, both showed negative staining. (D) is BAVM tissue, showed positive staining of brain parenchyma. (E and F) are BAVM tissues which showed positive staining of MMP-9 in the endothelial cells (black arrow) and perivascular cells (red arrow). A total of 16 BAVM tissues were stained with anti-MMP-9. Original magnification ×100 (A, B) and ×200 (C–F).

We analyzed the expressions of the candidate proteins OPN, MMP-2 and -9 in 16 BAVM cases as summarized in Table 1. Immunohistochemical staining with OPN, MMP -2 and -9 were seen in brain parenchyma, endothelial cells and perivascular cells of BAVM patients while negative staining was observed in negative controls and normal brain specimens (Figs. 2–4). All BAVM tissues (16; 100%) were positive for OPN and MMP2 proteins and amongst these, 13 (81%) were positive for MMP-9 proteins. Moderate positivity (++) of OPN protein expression was detected in most BAVM tissues (13; 81%). There were six BAVM tissues showed high positivity (+++) of either MMP-2 or -9 proteins, indicating a negative trend between MMP-2 and -9 expressions although this did not achieve formal statistical significance (*p* = 0.321; Fisher’s exact test).

**Table 1:** Summary of immunohistochemical analysis of expression of OPN, MMP-2 and -9 proteins in BAVM tissues.

Case No	Age	OPN	MMP-2	MMP-9
BAVM 1	42	++	+	+++
BAVM 2	12	++	++	+
BAVM 3	56	++	+	Neg
BAVM 4	29	++	+	Neg
BAVM 5	11	++	++	Neg
BAVM 6	27	++	+++	++
BAVM 7	67	+	+	+
BAVM 8	18	++	++	++
BAVM 9	36	++	++	+
BAVM 10	29	++	+	+
BAVM 11	16	+	+++	++
BAVM 12	53	++	++	+++
BAVM 13	21	++	++	+++
BAVM 14	28	++	++	+++
BAVM 15	56	++	++	++
BAVM 16	38	+	+	+

**Note:**

Staining score describes the proportion of positive cells; +++ (80%), nearly all cell stained; ++ (50), approximately half of the cell stained; + (10%), a low percentage of cell stained; Neg, no positive cell detected.

In Fig. 2A, is a colonic tissue (positive control) which showed positive staining in the intestinal glands. Figs. 2B and 2C are normal brain tissue with antibody and BAVM tissue without antibody, respectively, as negative controls, both showed negative staining for OPN. Fig. 2D is BAVM tissue stained with OPN, showed positive staining the of the brain parenchyma. Figs. 2E and 2F are also BAVM tissues, showing positive staining of OPN in the endothelial cells (black arrow) and perivascular cells (red arrow), respectively.

Fig. 3A is a section of neurofibroma (as positive control), which showed positive staining of MMP-2 in the neural elements. Figs. 3B and 3C are normal brain tissue with antibody and BAVM tissue without antibody, respectively, as negative controls, both showed negative staining. Fig. 3D is BAVM tissue, showed positive staining in the brain parenchyma. Figs. 3E and 3F are also BAVM tissues, both of which showed positive staining of MMP-2 in endothelial cells (black arrow) and perivascular cells (red arrow).

In Fig. 4A, is liver tissue (as positive control) which showed positive staining of MMP-9 in the hepatocytes. Figs. 4B and 4C are normal brain tissue with antibody and BAVM tissue without antibody, respectively, as negative controls, both showed negative staining. Fig. 4D is BAVM tissue, showed positive staining of brain parenchyma. Figs. 4E and 4F are BAVM tissues which showed positive staining of MMP-9 in the endothelial cells (black arrow) and perivascular cells (red arrow).

## Discussion

Brain AVM is often an asymptomatic vascular disease until the onset of ICH which can cause death (Kim et al., 2011; Komiyama, 2016). Early detection of BAVM is possible when patients present with symptoms such as progressively worsening headache or sudden onset of seizure, and subsequently they undergo a brain scan (magnetic resonance imaging-MRI) (Stapleton et al., 2011). While the AVM may be confirmed through brain scans, analysis of the systemic levels of inflammatory markers associated with BAVM may help in predicting the severity of the lesions and risk of rupture. In this study, we investigated the inflammatory markers, OPN, MMP-2 and -9 in the serum of BAVM patients before and after surgery or/and embolization. We also studied the localization of these markers in tissue samples of BAVM patients and compared with normal brain samples.

In a study, researchers used white pigs to evaluate the in vivo presentation of OPN resulting from vein grafting, and their results suggested that the expression of OPN significantly increased postoperatively in the intima of the vein grafts in the first week and that from the second week onward OPN expression gradually declined. This study also showed that the expression of MMP-2 and -9 were correlated well with the expression of OPN (Kang et al., 2012). This suggests that expression of OPN, MMP-2 and -9 is able to show the alteration of expression in tissue within 2 weeks of surgery. Based on this observation, post-operative blood collection in our study was conducted 2 weeks after surgery to analyze the difference in serum OPN, MMP-2 and -9 levels before and after surgery. Furthermore, clinically, patients who had undergone surgical intervention are routinely assessed after 2 weeks in our institution, by which time the symptoms of brain AVM had disappeared and the healing of their surgical wounds are in progress.

Matrix metalloproteinases are involved in matrix degradation, tissue remolding around both normally as well as abnormally developing vasculature and mediate early inflammatory processes (Costanzo & Perrino, 2008; Feiler et al., 2011). In this study, we observed significant increase in MMP-2 and -9 levels in the serum of BAVM patients before and after surgery or/and embolization, comparing to control serums. MMP-2 and -9 expression was also observed in BAVM tissues. Expressions of MMP-2 and -9 in both serum and tissue samples of BAVM patients could be due to extracellular degradation of vascular matrix in the existence of vascular instability in BAVM patients. It has been postulated that MMP-2 and -9 activation enabled the degradation of extracellular matrix component, causing damage to the stability and integrity of blood vessels, weakening the blood brain barrier and resulting in increased risk of hemorrhage (Yang et al., 2007). However, some of the BAVM tissues showed negative staining for MMP-9. This variation in staining pattern could be due to the yet undetermined origin and unpredictable natural progression, as each patient’s clinical behavior and end point are known to be different in BAVM (Xu et al., 2012). The elevated levels of MMP-2 and -9 expression in the serum of BAVM patients after the surgery or/and embolization compared to control samples could be due to angiogenesis that takes place in wound healing process. This generates blood vessel-rich granulation tissue, a critical step in tissue regeneration. During wound healing, MMP-2 and -9 are involved in the regulation of angiogenesis by activating the pro-angiogenic cytokines, including TNF-α and VEGF, and by generating antiangiogenic peptides such as endostatin from type XVII collagen, the latter of which is expressed in the basement membrane (Caley, Martins & O’Toole, 2015). Further analysis of serum MMP-2 and -9 levels should be carried out for an extended time point after treatment to monitor whether there will be a decline after the wound has healed compared with pre-treatment serum levels or to determine if the expression of MMP-2 and -9 remains high in BAVM patients post-treatment.

Our investigation of OPN showed that there is no difference in the OPN levels between BAVM patients’ serums and control serums before and after surgery. Also, serum OPN levels were not detected in BAVM patients and controls before and after surgery. A large sample size is needed to validate this finding. OPN was found to be focally overexpressed in endothelial cells of BAVM tissues, supporting the documented evidence in previous studies on OPN IHC in BAVM tissue and the involvement of OPN in vascular remolding process and physiological response towards the hemodynamic stress arises from increased pressure in arteriovenous shunting which weakens the vessels (Xu et al., 2012).

Studies have also reported that OPN acts as substrate for several MMPs including MMP-2, MMP-3, MMP-7 and MMP-9 (Agnihotri et al., 2001; Dean & Overall, 2007; Takafuji et al., 2007). Cleavage of OPN by MMPs occurs at several sites on the OPN molecule. The cleaved form of OPN possesses more potent activity than the full-length form (Agnihotri et al., 2001). However, in this study, a disproportionate expression of OPN and MMPs was observed, where the serum level of OPN was negligible while serum MMP-2 and -9 levels were elevated. Besides that, IHC showed that the expression of OPN, MMP-2 and -9 was localized mainly on the vessel walls. This finding lends support to the postulation that these markers play a role in the weakening of vessel walls. Further analysis of these markers in tissue samples is required to study the pathogenesis of instability of vessel walls, perhaps in vitro experiments involving the knockdown of the expression of OPN, MMP-2 and -9.

## Conclusion

This preliminary study on the inflammatory markers involved in the destabilization of blood vessels showed the expression of OPN, MMP-2 and -9 in tissue samples of BAVM patients. The localization of the proteins is mainly on the lining of the blood vessels. This confirms the involvement of OPN, MMP-2 and -9 in pathophysiology of the vascular destabilization in BAVM. Furthermore, serum analysis shows the elevation of MMP-2 and -9 in BAVM patients compare to controls. However, OPN expression is not observed in serum samples of BAVM patients. Larger sample sizes are needed to validate this finding. In some cases, MMP-9 expressions were found to be negative. This could be due to the undetermined origin and unpredictable natural progression of the disease, as each patient’s clinical behavior and end point are known to be different in BAVM. In several cases, the level of MMP-2 and -9 in patients’ serum co-related well with their expression in BAVM tissue. Knockdown studies need to be carried out to study the relationships and mechanisms of action of these markers to investigate if the expression of these markers could help in predicting the rupture and in identifying the primary medical therapy targets in BAVM patients.

## Limitation

The number of recruited samples for serum analysis is low because BAVM is rare. Furthermore, only some cases will be treated with embolization and surgery. Most of the BAVM patients will be under observation and not given any treatment. In our study, we recruited patients with BAVM who have underwent the treatments, embolization or/and surgery. Therefore, the number of patients recruited for serum analysis from 2014 to 2016 is less than 10. A larger sample is needed to validate the serum analysis, especially for OPN since the protein is expressed in tissue samples.

## Supplemental Information

10.7717/peerj.7058/supp-1 Supplemental Information 1 OPN ELISA assay raw data. The raw data shows the reading of standards, blanks and samples. The were duplicates for each samples. The average absorbance readings were calculated and the concentrations were taken for each samples through the graph.

10.7717/peerj.7058/supp-2 Supplemental Information 2 MMP-2 Multiplex ELISA assay raw data. The raw data shows the reading of standards, blanks and samples. The were duplicates for each samples. The average absorbance readings were calculated and the concentrations were taken for each samples through the graph.

10.7717/peerj.7058/supp-3 Supplemental Information 3 MMP-9 Multiplex ELISA assay raw data. The raw data shows the reading of standards, blanks and samples. The were duplicates for each samples. The average absorbance readings were calculated and the concentrations were taken for each samples through the graph.

10.7717/peerj.7058/supp-4 Supplemental Information 4 OPN, MMP-2 and -9 tables for box plot. Tables of OPN, MMP-2 and -9 to generate box plots.

## Additional Information and Declarations

### Data Availability

The following information was supplied regarding data availability: The raw data is available as Supplemental Files.
